# Reduced Carotenoid and Retinoid Concentrations and Altered Lycopene Isomer Ratio in Plasma of Atopic Dermatitis Patients

**DOI:** 10.3390/nu10101390

**Published:** 2018-10-01

**Authors:** Renata Lucas, Johanna Mihály, Gordon M. Lowe, Daniel L. Graham, Monika Szklenar, Andrea Szegedi, Daniel Töröcsik, Ralph Rühl

**Affiliations:** 1 Department of Dermatology, Faculty of Medicine, University of Debrecen, 4032 Debrecen, Hungary; renata.lucas@rocketmail.com (R.L.); aszegedi@med.unideb.hu (A.S.); dtorocsik@gmail.com (D.T.); 2 Department of Biochemistry and Molecular Biology, University of Debrecen, 4032 Debrecen, Hungary; johanna@med.unideb.hu; 3 School of Pharmacy and Biomolecular Sciences, Liverpool John Moores University, Byrom Street, Liverpool L3 3AF, UK; g.m.lowe@ljmu.ac.uk (G.M.L.); D.L.Graham@ljmu.ac.uk (D.L.G.); 4 Faculty of Science, Liverpool John Moores University, Byrom Street, Liverpool L3 3AF, UK; 5 Paprika Bioanalytics BT, 4002 Debrecen, Hungary; monikaszklenar1@gmail.com

**Keywords:** lycopene, carotene, retinoic acid, retinoid, vitamin A, RAR, RXR

## Abstract

Carotenoids and retinoids are known to alter the allergic response with important physiological roles in the skin and the immune system. In the human organism various carotenoids are present, some of which are retinoid precursors. The bioactive derivatives of these retinoids are the retinoic acids, which can potently activate nuclear hormone receptors such as the retinoic acid receptor and the retinoid X receptor. In this study, we aimed to assess how plasma carotenoid and retinoid concentrations along with the ratio of their isomers are altered in atopic dermatitis (AD) patients (*n* = 20) compared to healthy volunteers (HV, *n* = 20). The study indicated that plasma levels of the carotenoids lutein (HV 198 ± 14 ng/mL, AD 158 ± 12 ng/mL, *p* = 0.02; all values in mean ± SEM), zeaxanthin (HV 349 ± 30 ng/mL, AD 236 ± 18 ng/mL, *p* ≤ 0.01), as well as the retinoids retinol (HV 216 ± 20 ng/mL, AD 167 ± 17 ng/mL, *p* = 0.04) and all-*trans*-retinoic acid (HV 1.1 ± 0.1 ng/mL, AD 0.7 ± 0.1 ng/mL, *p* = 0.04) were significantly lower in the AD-patients, while lycopene isomers, α-carotene, and β-carotene levels were comparable to that determined in the healthy volunteers. In addition, the ratios of 13-*cis*- vs. all-*trans*-lycopene (HV 0.31 ± 0.01, AD 0.45 ± 0.07, *p* = 0.03) as well as 13-*cis*- vs. all-*trans*-retinoic acid (HV 1.4 ± 0.2, AD 2.6 ± 0.6, *p* = 0.03) were increased in the plasma of AD-patients indicating an AD-specific 13-*cis*-isomerisation. A positive correlation with SCORAD was calculated with 13-*cis*- vs. all-*trans*-lycopene ratio (*r* = 0.40, *p* = 0.01), while a negative correlation was observed with zeaxanthin plasma levels (*r* = −0.42, *p* = 0.01). Based on our results, we conclude that in the plasma of AD-patients various carotenoids and retinoids are present at lower concentrations, while the ratio of selected lycopene isomers also differed in the AD-patient group. An increase in plasma isomers of both lycopene and retinoic acid may cause an altered activation of nuclear hormone receptor signaling pathways and thus may be partly responsible for the AD-phenotype.

## 1. Introduction

Carotenoids and retinoids are considered to have beneficial effects in the prevention of many major diseases and they play a crucial role in skin physiology and allergic responses [1,2,3,4,5,6,7,8,9,10,11]. Retinoids regulate a variety of physiological processes, such as proliferation, differentiation, immune regulation, and epidermal barrier function [12]. The term retinoids include both natural forms of vitamin A, retinaldehyde, and retinoic acid, as well as synthetic retinol analogs. A high vitamin A or high pro-vitamin A containing diet resulted in increased plasma levels of all-*trans* retinoic acid thus providing an important association between nutritional factors and retinoic acid signaling mediated pathways [13]. Several skin diseases have been associated with alterations of the retinoid metabolism and signaling [14].

Vitamin A and retinoid derivatives play a pivotal role in cutaneous physiology, and various skin diseases have been associated with altered retinoid metabolism and signaling, such as atopic dermatitis (AD), which is a common chronic inflammatory skin disease, showing structural abnormalities of the epidermal barrier, and is characterized by increased IgE secretion and Th2 response [9,14,15,16,17,18]. Bioactive retinoic acids activating RXR and RAR mediated signalling are also affecting various aspects concerning an allergic skin inflammation like a systemic Th1/Th2 shift [9,17,18] as well as a topical allergic skin inflammation [2,3,19,20].

Carotenoids, a family of more than 600 compounds, are important micronutrients in the human diet and are also present in the human plasma [10,21,22]. The major and most studied of the dietary carotenoids are β-carotene, lycopene, lutein, and zeaxanthin. β-Carotene is known to have the greatest pro-vitamin A activity [23]. Lycopene, a red pigment mainly originating from tomatoes and tomato products, is found as all-*trans* isomer in the majority of the food sources [24]. *Cis*-isomers of lycopene are also found in biological systems with the 5-*cis*, 9-*cis*, 13-*cis*, and 15-*cis* isomers being the most predominant forms [25]. Lycopene is also present in human and animal tissues, but it is found mainly as *cis*-isomers, of which the 5-*cis* isomer is the most predominant form [26,27]. Unfortunately, except for β-carotene and partly lycopene functioning as known or potential precursors for RAR and RXR ligands [28,29,30], no clearly defined mechanisms of action has been found, except the controversial discussed and partly non nutritional and physiological relevant antioxidant potential [31].

Supporting the importance of retinoic acid in AD (patho)-physiology, in our previous study, we showed that retinoic acid levels are lower in the skin of AD-patients in comparison to healthy volunteers. Based on the observed alterations in retinoid transport, synthesis and plasma concentrations we concluded that retinoid signaling pathways might contribute to AD pathogenesis [2,3,11,32], which is also confirmed by numerous studies in rodents [1,6,33]. In this study, we aimed to assess whether the concentrations of carotenoids and retinoids, especially lycopene and its isomers along with retinoic acid and its isomers, which are potential indirect or direct activators of the RAR- and RXR-mediated signalling pathways, differ in the plasma of healthy volunteers and AD-patients.

## 2. Materials and Methods

**Study population:** After informed consent and the approval of the local Ethics Committee of the University of Debrecen, Hungary, Medical, and Health Science Centre, peripheral blood was collected from 20 AD-patients (8 male, 12 female; mean age 20 years, range 15–32 years). A group of 20 healthy age-matched volunteers (six males, 14 females, mean age 21 years, range 19–24 years) served as controls in this study. All AD patients fulfilled the diagnostic criteria established by Hanifin and Rajka [34].

The severity and activity of the disease was determined by the SCORAD (SCORe Atopic Dermatitis) index [35] and in our AD-patients the mean SCORAD was 35.2 (range 13–64). Patients were additionally tested for plasma total IgE by ELISA (ADALTIS Italia S.p.A., Casalecchio di Reno, Italy) according to the manufacturer’s instructions. The white cell count along with the absolute count of eosinophils in whole blood was determined while using an Advia 120 haematology analyzer (Siemens, München, Germany).

The plasma samples that were used in this study originate of a larger pool of plasma that was obtained from previous published studies and where different lipid profiles were analysed [36,37,38]. In eight of the 20 patients the disease started in the first year of life, in two patients between ages 3–4, in seven patients between 6–18 years, and in three patients in adulthood (>18 years). In patients’ history, 10 patients of 20 had rhinitis, three had asthma, and three had both rhinitis and asthma (Table 1). Patients, have not been treated with oral glucocorticosteroids, non-steroidal anti-inflammatory drugs or other systemic immunomodulatory agents for at least four weeks, also did not receive antihistamins and topical corticosteroids for at least five days prior to blood sampling.

**Plasma sample preparation:** Peripheral blood was collected into EDTA-containing BD vacutainer blood collection tubes (Becton-Dickinson, BD Diagnostics, Le Pont de Claix, France) and transferred to a 15 mL falcon tube (Sigma Aldrich, Budapest, Hungary) immediately after collection and then centrifuged under dimmed yellow light at room temperature, 2500 rpm for 15 min. Plasma was removed after centrifugation and kept on −80 °C.

**HPLC analysis for carotenoids:** The plasma samples were obtained in Debrecen and transported on dry ice and exclusion of light to Liverpool John Moores University, for analysis. Upon arrival the samples were stored at −80 °C until they were processed.

Carotenoids were extracted from plasma samples using the following method: 1.0 mL aliquot of patient’s plasma was added to a glass flip-top squat vial and 1.0 mL of ethanol added. The sample was vortexed immediately for 2 s, prior to the addition of 1.5 mL diethyl ether. The sample was vortexed again for 2 s prior to the addition of 1.5 mL hexane. The sample was then vortexed a last time for 2 s and then allowed to stand for partition. The top layer of the sample was then removed with a glass Pasteur pipette and dried down in a 4 mL amber screw-top vial under oxygen free nitrogen conditions. The dry sample was further resuspended in 100 µL tetrahydrofuran and 400 µL methanol and then transferred to an amber 2 mL HPLC vial prior to analysis.

All HPLC was performed on an Agilent 1100 series fully automated HPLC (Agilent Technologies UK Ltd., Berkshire, UK) with diode array detection. All samples were analysed using either a C18 or C30 column (VWR International Ltd., Lutterworth, UK). C18 column was used for estimating the concentration of carotenoids (β-carotene, lycopene, β-cryptoxanthin, lutein) [39] and the C30 was used for determining the concentration of isomers for β-carotene and lycopene. Concentrations were estimated via comparison with purified external standard samples under identical HPLC conditions [40,41].

The precision of carotenoid analysis in plasma samples was determined ahead of the analysis of our examined samples. The *intra*-day precision was determined based on *n* = 12 consecutive analysis of the same human plasma sample under the same separate extraction and analysis conditions and it resulted in %-coefficient of variation (%CV) = 7.7 for all-*trans* lycopene (determined concentration of 0.65 ± 0.05 µmol/L) and %CV = 7.5 for β-carotene (determined concentration of 0.53 ± 0.04 µmol/L).

The *inter-*day precision of the carotenoid analysis was determined using the same pooled samples which were previously aliquoted and individually stored at −80 °C. Each individual consecutive day (*n* = 10) ahead of the analysis these aliqots were defrosted, freshly extracted and analyzed each day (*n* = 10). We determined a %CV = 8.8 for all-*trans* lycopene and %CV = 9.1 for β-carotene.

**Analysis using a C18 reverse phase column:** From our plasma aliquot, a sample volume of 50 µL was injected into the HPLC using an auto-sampler. The solvent system used was 66/22/10 acetonitrile/tetrahydrofuran/methanol (0.005% *w*/*v* ammonium acetate). The solvent mixture was delivered at a rate of 0.8 mL/min. The column was held at a temperature of 22 °C. The column was protected using a C18 guard column supplied by Phenomenex (Macclesfield, UK). Lycopene and its components were separated using a 5 µm Gemini (Phenomenex, Macclesfield, UK) C18 reverse phase column (4.6 × 250 mm).

**Analysis using a C30 column:** A sample volume of 50 µL was injected into the HPLC while using an auto-sampler. The solvents system comprised of 50/40/10 methyl-tert-butylether/methanol/ethyl acetate. This was delivered isocratically at a rate of 0.45 mL/min. The column was a YMC (VWR, Lutterworth, UK) C30 (5 µm 4.6 × 250 mm), and kept at a temperature of 40 °C. The column was protected using a C30 guard column that was supplied by YMC (VWR, Lutterworth, UK).

The run times for the C18 column was 20 min, whilst for the C30 column, it was 35 min. The detection of the eluted compounds was by diode array screening between 300–600 nm and integration of each peak was performed using the Chemstation software (v10A) (Agilent Technologies UK Ltd., Berkshire, UK).

**HPLC MS-MS analysis for retinoids:** Concentrations of 13CRA, ATRA and retinol were determined in human plasma samples by our high performance liquid chromatography mass spectrometry—mass spectrometry (HPLC MS-MS) method as described previously [42]. In draft, high performance liquid chromatography mass spectrometry (2695XE separation module; Waters, Waters, Budapest, Hungary)—mass spectrometry (Micromass Quattro Ultima PT; Waters, Budapest, Hungary), analyses were performed under dark yellow/amber light while using previously validated protocol. For sample preparation, 100 μL plasma was diluted with a threefold volume of isopropanol vortexed for 10 s, put in an ultra-sonic bath for 5 min, shaken for 6 min, and centrifuged at 13,000 rpm in a Heraeus BIOFUGE Fresco at +4 °C. After centrifugation, the supernatants were dried in an Eppendorf concentrator 5301 (Eppendorf, D) at 30 °C. The dried extracts were resuspended with 60 μL of methanol, diluted with 40 μL of 60 mM aqueous ammonium acetate solution and transferred into the autosampler and subsequently analyzed. Quantification was performed like previously described in [42].

**Statistics:** The data comparing healthy volunteers and AD-patients are shown as mean and standard error mean (SEM) based on 2 *x n* = 20 samples using a paired student’s *t*-test analysis while considering a *p* value of less than 0.05 significant. Statistical analysis was performed using Graph pad Prism (version 7.04 for windows; GraphPad Software, Inc., LaJolla, CA, USA).

**Figure 1:**
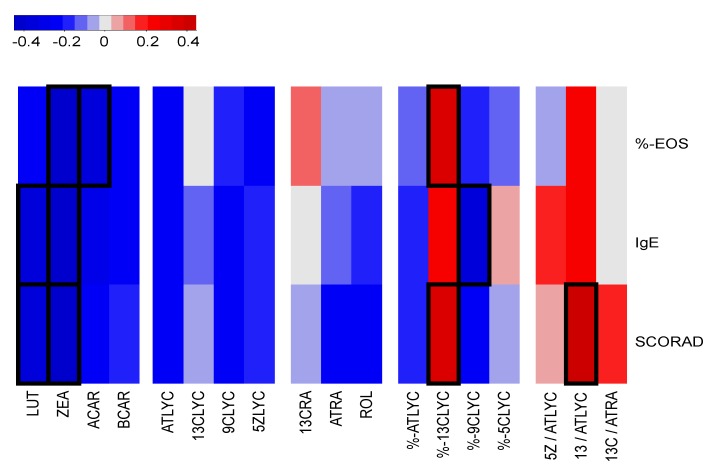
In this clustered image map analysis individual correlations between two values are displayed based on the determined concentrations and calculated ratios and percentile amounts originating from *n* = 40 individuals (healthy volunteers and AD-patients). A specific correlation value is indicated by a two-dimensional colored image (red to blue), where each entry of the matrix is colored on the basis of its correlation value indicated by the displayed color legend in a distance matrix analysis from plasma levels of the carotenoids zeaxanthin (ZEA), lutein (LUT), β-carotene (BCAR), α-carotene (ACAR), all-*trans*-lycopene (ATLYC), 13-*cis*-lycopene (13CLYC), 9-*cis*-lycopene (9CLYC), 5-*cis*-lycopene (9CLYC) and the retinoids 13-*cis*-retinoic acid (13CRA), all-*trans*-retinoic acid (ATRA), retinol (ROL) levels in addition to calculated ratios and percentile amounts of selected carotenoids or retinoids with the clinical AD-markers, like number of eosinophils as percentage from PBMCs (%-EOS), plasma total IgE levels in kilounits per liter (IgE), and SCORAD units [45,46]. Significant correlations are indicated by a *p* > 0.05 and were additionally marked by a black frame.

For the correlation analysis displayed in Figure 1 and Figure S1 data from *n* = 40 individuals, healthy volunteers and AD-patients were combined, and Spearman correlation (*r*- and *p*-value) were calculated in “R” 3.3.2. version to determine the relationship between the carotenoid and retinoid levels as well as calculated percentile amounts and ratios with the three clinical AD-markers (IgE, %-EOS and SCORAD). The correlation analysis and heap map creation was also performed in “R” 3.3.2. version via the “heatmap.2” function [43]. Significance indicated by a *p*-values > 0.05 were marked with a black frame in Figure 1.

## 3. Results

### 3.1. Characterisation of the Study Cohort

**Characterisation of the study cohort:** The same patient cohort was used as in our previous studies [36,37,38]. AD-patients and healthy volunteers did not differ in age and gender. Clinical markers of atopic dermatitis (AD) like SCORAD [35] and ongoing discussion about validity as an AD marker [44], total IgE, and percental amount of eosinophils from peripheral blood mononuclear cells (PBMCs) were significantly increased (Table 1).

### 3.2. Carotenoid Concentrations and Lycopene Isomers

**Reduced concentrations of lutein and zeaxanthin in AD-patients:** Lutein and zeaxanthin plasma levels were significantly decreased in the plasma of AD patients compared to healthy volunteers (from 198 ± 14 ng/mL to 158 ± 12 ng/mL, *p* = 0.02, respectively, from 349 ± 30 ng/mL to 236 ± 18 ng/mL, *p* > 0.01). α-Carotene and β-carotene levels were comparable in healthy volunteers and AD-patients (Table 2A).

**Altered lycopene isomer concentrations in healthy volunteers and AD-patients:** Total lycopene levels display a non-significantly trend to be lower in the plasma of AD-patients (from 281 ± 30 ng/mL to 248 ± 160 ng/mL). Individual concentrations of lycopene isomers, like all-*trans* (from 126 ± 15 ng/mL to 107 ± 17 ng/mL), 9-*cis* (from 25 ± 3 ng/mL to 21 ± 3 ng/mL), and 5-*cis* (from 94 ± 10 ng/mL to 80 ± 12 ng/mL), isomers also showed a non-significant trend of lower levels in the plasma of AD-patients, while the concentration of 13-*cis*-lycopene displayed a non-significant trend of lower levels when compared to healthy volunteers from 36 ± 3 ng/mL to 40 ± 3 ng/mL (Table 2B).

**13-*cis*-lycopene%-amounts of lycopene isomers show significant alterations in AD-patients compared to healthy volunteers:** The 13-*cis* lycopene ratio has been significantly increased (from 13.3 ± 0.4% to 17.2 ± 1.7%). All-*trans*-, 9-*cis*- and 5-*cis*-lycopene did not show significant alterations in their %-amounts (Table 2C).

**Calculated ratios of selected lycopene isomers in healthy volunteers and AD-patients:** 13-*cis-*/all-*trans-*lycopene ratios has been significantly increased in AD patients as compared to healthy volunteers from 0.31 ± 0.01 to 0.45 ± 0.07 (*p* = 0.03), while no alteration could be observed in the 5-*cis-*/all-*trans*-lycopene ratio (from 0.78 ± 0.03 to 0.79 ± 0.04) (Table 2D).

### 3.3. Retinoid Concentration and Ratios of Retinoic Acid Isomerization

**Reduced retinoic acid and retinol concentrations in AD-patients compared to healthy volunteers:** All-*trans*-retinoic acid concentrations were significantly lower in the plasma of AD-patients (0.7 ± 0.1 ng/mL) compared to healthy volunteers (1.1 ± 0.1 ng/mL, *p* = 0.04), while 13-*cis*-retinoic acid concentrations just display a non-significant trend of lower levels (from 1.2 ± 0.1 ng/mL to 1.0 ± 0.1 ng/mL). Retinol concentrations were also significantly decreased in plasma of AD-patients when compared to healthy volunteers (from 216 ± 20 ng/mL to 167 ± 17 ng/mL, *p* = 0.04). Our results showed that both ATRA and retinol were present in a lower concentration in the plasma of atopic individuals (Table 3A).

**Ratio of plasma levels of retinoic acid isomers 13CRA/ATRA:** The 13CRA/ATRA ratio was significantly increased in the plasma of AD-patients from 1.4 ± 0.2 to 2.6 ± 0.6 (*p* = 0.03) (Table 3B).

### 3.4. Correlation Analysis

**Zeaxanthin levels negatively and 13*-cis-*/all*-trans-*lycopene ratios positively correlate to clinical AD-markers:** The correlation analysis between plasma values and calculations of percentile amounts and ratios of selected retinoids and carotenoids originating from *n* = 40 individuals (healthy volunteers and AD-patients) determined a significant positive correlation of plasma 13*-cis-*/all*-trans-*lycopene ratios (*r* = 0.40, *p* = 0.01) with SCORAD. A negative correlation of plasma lutein levels with SCORAD (*r* = −0.36, *p* = 0.02) and IgE (*r* = −0.33, *p* = 0.04), a negative correlation with plasma zeaxanthin levels with %-EOS (*r* = −0.41, *p* = 0.01), IgE (*r* = − 0.45, *p* < 0.01), and SCORAD (*r* = −0.42, *p* = 0.01).

Further positive or negative correlation with plasma levels of ACAR with %-EOS (*r* = −0.35, *p* = 0.02), %-13CLYC with %-EOS (*r* = 0.33, *p* = 0.04) and SCORAD (*r* = 0.34, *p* = 0.03), and 9CLYC with IgE (*r* = −0.34, *p* = 0.03) were calculated. No further significant correlations were found with the clinical AD-markers number of eosinophils as percentage from PBMCs (%-EOS) or plasma total IgE levels in kilounits per liter (IgE) (outlined in Figure 1).

In addition, we did not observe any significant correlation between plasma ATBC and ATRA levels of *n* = 40 individuals (healthy volunteers and AD-patients) (Figure S1).

## 4. Discussion

Based on analysis of retinoids and carotenoids in human plasma, as well as calculating relevant ratios, we observed that specific carotenoids, like zeaxanthin and lutein, as well as the retinoids retinol and all-*trans*-retinoic acid (ATRA) are lower in plasma of AD-patients vs healthy individuals. Additionally, we observed that the %-amount of 13-*cis*-isomers based on calculated ratios of lycopene and retinoic acid isomers were higher in AD-patients. If these found alterations are a cause or a consequence of AD needs to be discussed and further examined.

Naturally occurring forms of vitamin A and other synthetic retinoid analogues are mainly present in the all-*trans*-configuration form, but *cis-*isomers also have relevant biological roles, maintaining essential physiological processes in the human organism, such as vision, cellular growth and differentiation, reproduction, normal growth and development, healthy immune system, and healthy skin and barrier functions [45,46]. Increased ATRA concentrations have been shown to increase retinoic acid-mediated signaling via RAR- and RXR-mediated signalling pathways. Surprisingly, in this study calculating the individual correlation between the plasma values of ATRA with plasma levels of the main pro-vitamin A all-*trans*-β-carotene (ATBC, Figure S1) displayed no significant correlation determined in all individuals (*n* = 40, healthy adults and AD-patients). Therefore, the connection between increased carotenoid intake with following increased plasma ATBC levels and further increased RAR- and RXR-mediated signalling seems to be questionable, like discussed and co-found in recent reports [4,23].

The retinoic acid exists in three major stereoisomeric forms: (ATRA), 9-*cis*-retinoic acid (9CRA), and 13*-cis*-retinoic acid (13CRA, also known as isotretinoin) [47]. ATRA binds only to retinoic acid receptors (RARs), while 9CRA can bind to both RARs and retinoid X receptor (RXRs). Recently the real endogenous RXR ligand 9-*cis*-13,14-dihydroretinoic acid was identified by our group [48,49]. The 13-*cis-*isomer of retinoic acids does not bind specifically to RXRs and it has a lower affinity to RARs then ATRA or 9CRA [50]. In general, 13CRA is considered to be a non active form of the biologically active ATRA and it is generated endogenously, non-enzymatically by spontaneous isomerization from ATRA [51], or enzymatically by means of a novel identified enzyme 13-*cis* specific isomerohydrolase, which generates exclusively 13-*cis*-retinol, a precursor of 13CRA [52]. A reduced, non-enzymatic or enzymatic, isomerization back to the all-*trans* configuration maybe an alternative reason of this altered isomer-distribution occurring in serum of atopic dermatitis patients [53,54]. This retinoid- and carotenoid-isomerization is still a highly controversial topic and multiple mechanisms might occur.

Lycopene isomers were analyzed as well as %-amounts of lycopene isomers all*-trans-*, 13-*cis-,* 9-*cis-* and 5-*cis*-lycopene were calculated and we found significantly increased %-amounts of 13-*cis*-lycopene in plasma of AD-patients as well as an increased ratio of 13-*cis*-/all-*trans*-lycopene compared to healthy volunteers. We conclude that a specific 13-*cis* isomerization of retinoic acid and of lycopene might likely be a consequence of the AD-phenotype. The 13-*cis-*/all-*trans-*lycopene ratio positively correlated with the clinical AD-marker SCORAD. This indicates that neither the systemic AD-markers IgE and %-EOS, but more likely the skin with the specific AD-marker SCORAD, might be co-involved with that specific 13-cis-isomerization. It stays still elusive, which cells, which specific enzymes and which alternative specific 13-*cis*-isomerization inducing mechanisms are behind this phenomenon.

In our examined AD-patients, lutein and zeaxanthin concentrations were also significantly lower in the plasma compared to healthy volunteers and both negatively correlated with the AD-markers SCORAD, IgE and %-EOS (excluding lutein) as visualized in Figure 1. This let us postulate potential positive effects of food rich in zeaxanthin on AD. As serum levels of lutein and zeaxanthin are increased by dietary intake of food rich in fruits and vegetables [55,56] as well as supplements rich in zeaxanthin and lutein [57] a potential connection between a healthy diet and AD can be postulated [58]. Recently, positive effects of lutein and zeaxanthin supplementation and local administration of these carotenoids have been shown to be beneficial for the human skin [59,60]. The precise mechanism of action and a mechanistic explanation of this found phenomenon remain elusive. In addition, the reduced serum zeaxanthin levels may also be used as a biomarker and an indicator for AD.

Other carotenoids, like α-carotene, β-carotene, and lycopene were not altered in AD-patients, which is partly in agreement with a previous study in atopic children [7,61]. Unfortunately carotenoid levels in humans have a high variability due to altered individual nutrition, chronic inflammatory background, and genetic background [7,62,63,64]. A direct functional comparison of found alterations of carotenoid plasma levels of data originating from adults, examined this study, and children [7] cannot be done because the levels found in children were determined to be much lower. If these reduced levels of carotenoids in AD-patients or atopic children are based on higher chronic inflammation and a further pro-oxidant stress, a targeted selected downregulation of carotenoid absorption and binding, a different occurrence of carotenoid-levels specific polymorphisms or reduced intake of healthy food [21,23], remains speculative. One possible mechanism might be that lutein/zeaxanthin are known to negatively interfere with ATRA-RAR-mediated signalling [65,66,67] which was associated with pro-allergic Th2 immune responses [1,6,9,17,19].

Much larger cohorts examining carotenoid and retinoid levels, as well as genetic background, transcriptomic based regulation of these lipid pathways in children and adults also for multiple AD-relevant organs like the immune cells and healthy and affected skin should be planed and performed to get further knowledge about this phenomenon.

A special focus should be put on examining carotene oxygenases, which may cleave lycopene to yield retinoid-like derivatives [28,29,30], as still non-identified bioactive lycopene-metabolites. As retinoids have been shown to play important roles in skin homeostasis and signaling, alterations of retinoid signaling are related to several skin diseases and malignancies mediated by RARα, RARγ, RXR, and PPARδ-mediated signaling [2,3,68,69,70]. Altered retinoid concentrations, also based on potential novel lycopene-metabolites in the skin, might lead to altered nuclear hormone receptor pathway activation resulting in skin abnormalities and a further AD-specific phenotype.

We postulate that specific differences in retinoid or carotenoid isomerization via an enzymatic or non-enzymatic specific 13-*cis*-isomerisation in AD might be a consequence of chronic skin inflammation present in AD. These 13-*cis*/all-*trans* isomer ratios may have a still unknown biological meaning and may serve as a biological relevant priming factor for the AD-phenotype. Markers of 13-*cis*-isomerisation like the 13-*cis-*/all-*trans*-lycopene ratio positively correlates well with SCORAD and might also serve as a plasma biomarker for AD.

The limitations of the study are that the number of involved individuals was relatively low (*n* = 20), no food intake diaries were taken to evaluate differences in the dietary pattern of healthy volunteers and diseased patients in addition to skin/PBMC transcriptomics, retinoic/carotenoid profiling and following systems biology analysis, which would have resulted in a deeper advanced knowledge with a focus on the influence and connection of individual retinoids/carotenoids on AD.

## 5. Conclusions

Our study let us conclude that in the plasma of AD-patients various carotenoids and retinoids are present at lower levels, while the ratio of lycopene- and retinoic acid-isomers was also altered. These alterations might be a consequence of chronic skin inflammation that is present in AD and might cause an altered activation of nuclear hormone receptor signaling pathways that could be partly responsible for the AD-phenotype.

## Figures and Tables

**Table 1:** Clinical and basic demographic data from healthy volunteers and atopic dermatitis (AD)-patients, mean ± SEM.

	Healthy Volunteers	AD-Patients	*p*-Value
	*n* = 20	*n* = 20	*n* = 20
Age in years	21 ± 0.3	20 ± 1.2	0.48
Gender	70% female	60% female	-
SCORAD	0 ± 0	35.2 ± 4.3	**<0.01**
Total IgE (KU/L)	32 ± 0.0	2941 ± 1134	**0.01**
EOS %	2.5 ± 0	7.3 ± 1.1	**<0.01**

Total IgE—plasma total IgE levels in kilounits (KU)/L; SCORAD units [35], EOS %—number of eosinophils as percentage from PBMCs, AD-atopic dermatitis. This table is adapted from Mihaly et al., 2013 [38]. Numbers in bold letters indicate significance.

**Table 2:** Carotenoid concentrations (**A**), total sum and individual concentrations of lycopene isomers (**B**), calculated %-amounts of individual lycopene-isomers from the sum of lycopene isomers (**C**) and calculated ratios of selected lycopene isomers all calculated based on determined plasma concentrations from healthy volunteers and AD-patients.

(A) Carotenoid Concentrations			
in Plasma of Healthy Volunteers and AD-Patients.			
	Healthy Volunteers	AD-Patients	*p*-Value
	*n* = 20 (ng/mL)	*n* = 20 (ng/mL)	
lutein	198 ± 14	158 ± 12	**0.02**
zeaxanthin	349 ± 30	236 ± 18	**<0.01**
α-carotene	171 ± 21	149 ± 24	0.24
β-carotene	492 ± 77	394 ± 65	0.17
**(B) Total Sums and Concentration of Lycopene Isomers**			
**in Plasma of Healthy Volunteers and AD-Patients.**			
lycopene (sum)	281 ± 30	248 ± 35	0.24
lycopene (all-*trans*-)	126 ± 15	107 ± 17	0.20
lycopene (13-*cis*-)	36 ± 3	40 ± 5	0.29
lycopene (9-*cis*-)	25 ± 3	21 ± 3	0.17
lycopene (5-*cis*-)	94 ± 10	80 ± 12	0.19
**(C) Calculated %-Amounts of Lycopene Isomers**			
**in Plasma of Healthy Volunteers and AD-Patients.**			
	***n* = 20 (in%)**	***n* = 20 (in%)**	
lycopene (all-*trans*-)	44.1 ± 0.9	41.8 ± 1.3	0.08
lycopene (13-*cis*-)	13.3 ± 0.4	17.2 ± 1.7	**0.01**
lycopene (9-*cis*-)	8.8 ± 0.2	8.6 ± 0.3	0.24
lycopene (5-*cis*-)	33.8 ± 0.8	32.4 ± 1.2	0.16
**(D) Calculated Ratios of Selected Lycopene Isomers** **in Plasma of Healthy Volunteers and AD-Patients.**			
	***n* = 20 (Ratio)**	***n* = 20 (Ratio)**	
13-*cis*-/all-*trans*-lycopene	0.31 ± 0.01	0.45 ± 0.07	0.03
5-*cis*-/all-*trans*-lycopene	0.78 ± 0.03	0.79 ± 0.04	0.40

**Table 3:** (**A**) retinoic acid and retinol concentrations and (**B**) a calculated ratio of the plasma levels of retinoic acid isomers 13CRA/ATRA determined, based on plasma concentrations originating from healthy volunteers as well as AD-patients.

(A) Retinoic Acid and Retinol Concentrations in Human Plasma from Healthy Volunteers as well as AD-Patients.			
	Healthy Volunteers	AD-Patients	*p*-Value
	*n* = 20 (ng/mL)	*n* = 20 (ng/mL)	
ATRA	1.1 ± 0.1	0.7 ± 0.1	**0.04**
13CRA	1.2 ± 0.1	1.0 ± 0.1	0.17
ROL	216 ± 20	167 ± 17	**0.04**
**(B) Ratio of the Plasma Levels of Retinoic Acid Isomers 13CRA/ATRA.**			
	***n*** = **20 (Ratio)**	***n*** = **20 (Ratio)**	
13CRA/ATRA	1.4 ± 0.2	2.6 ± 2.6	**0.03**

Data are shown as mean and SEM based on *n* = 20 samples. ATRA—all-*trans*-retinoic acid, 13CRA—13-*cis*-retinoic acid, ROL—retinol. Numbers in bold letters indicate significance.

## Supplementary Materials

The following are available online at http://www.mdpi.com/2072-6643/10/10/1390/s1, Figure S1: Displayed is an MS-Excel calculated graphical direct correlation of plasma levels of all-*trans*-β-carotene (ATBC) and all-*trans*-retinoic acid (ATRA) plasma levels from *n* = 40 individuals (healthy volunteers and AD-patients). R- and *p*-values were previously calculated using “R” and were displayed graphically in Figure 1 and indicate no significant correlation between these two variables.
