# Supplementary material for: Reduced Carotenoid and Retinoid Concentrations and Altered Lycopene Isomer Ratio in Plasma of Atopic Dermatitis Patients
Source: Nutrients. 2018 Oct 1;10(10):1390. doi: 10.3390/nu10101390 (PMC6213761; doi:10.3390/nu10101390)
Supplement: Supplementary file 1 [file nutrients-10-01390-s001.pdf]

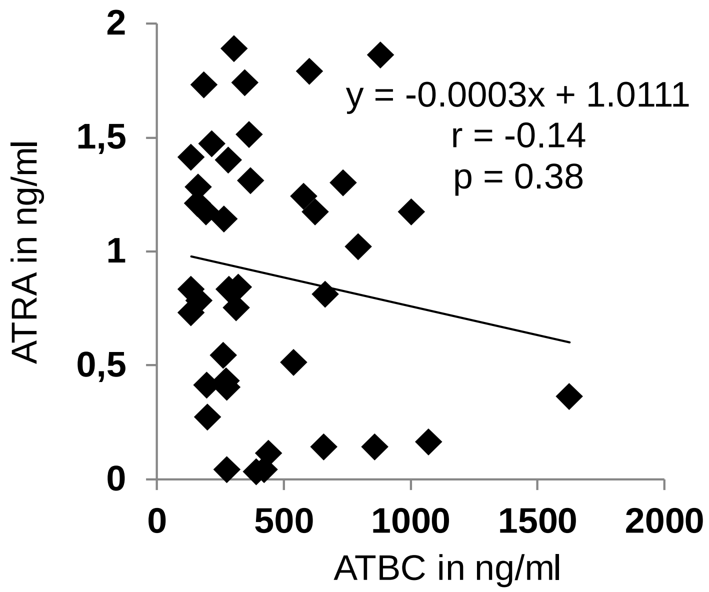

Supplementary Figure1: Displayed is an MS-Excel calculated graphical direct correlation of plasma levels of all-*trans*-β-carotene (ATBC) and all-*trans*-retinoic acid (ATRA) plasma levels from n = 40 individuals (healthy volunteers and AD-patients). R- and p-values were previously calculated using "R" and were displayed graphically in Figure 1 and indicate no significant correlation between these two variables.
